# GPS-Prot: A web-based visualization platform for integrating host-pathogen interaction data

**DOI:** 10.1186/1471-2105-12-298

**Published:** 2011-07-22

**Authors:** Marie E Fahey, Melanie J Bennett, Cathal Mahon, Stefanie Jäger, Lars Pache, Dhiraj Kumar, Alex Shapiro, Kanury Rao, Sumit K Chanda, Charles S Craik, Alan D Frankel, Nevan J Krogan

**Affiliations:** 1 Department of Cellular and Molecular Pharmacology, University of California San Francisco, 1700 4th Street, San Francisco, 94158 USA; 2 Department of Biochemistry and Biophysics, University of California San Francisco, 600 16th Street, San Francisco, 94158 USA; 3 Department of Pharmaceutical Chemistry, University of California San Francisco, 600 16th Street, San Francisco, 94158 USA; 4 Sanford-Burnham Medical Research Institute, 10901 North Torrey Pines Road, La Jolla, 92037 USA; 5 Immunology Group, International Centre for Genetic Engineering and Biotechnology, Aruna Asaf Marg, New Delhi 110 067, India; 6 TouchGraph LLC, 306 W. 92nd Street #3F, New York, 10025 USA

## Abstract

**Background:**

The increasing availability of HIV-host interaction datasets, including both physical and genetic interactions, has created a need for software tools to integrate and visualize the data. Because these host-pathogen interactions are extensive and interactions between human proteins are found within many different databases, it is difficult to generate integrated HIV-human interaction networks.

**Results:**

We have developed a web-based platform, termed GPS-Prot http://www.gpsprot.org, that allows for facile integration of different HIV interaction data types as well as inclusion of interactions between human proteins derived from publicly-available databases, including MINT, BioGRID and HPRD. The software has the ability to group proteins into functional modules or protein complexes, generating more intuitive network representations and also allows for the uploading of user-generated data.

**Conclusions:**

GPS-Prot is a software tool that allows users to easily create comprehensive and integrated HIV-host networks. A major advantage of this platform compared to other visualization tools is its web-based format, which requires no software installation or data downloads. GPS-Prot allows novice users to quickly generate networks that combine both genetic and protein-protein interactions between HIV and its human host into a single representation. Ultimately, the platform is extendable to other host-pathogen systems.

## Background

The application of high-throughput, unbiased, "systems" approaches to study host-pathogen relationships is facilitating a shift in focus from the pathogen to the response of the host during infection. A more global view of the physical, genetic and functional interactions that occur during infection will provide a deeper insight into the regulatory mechanisms involved in pathogenesis and may eventually lead to new cellular targets for therapeutic intervention.

Currently, the vast majority of host-pathogen physical interaction data involves HIV, for which a large amount of physical binding information has historically been available, mostly from small-scale, hypothesis-driven experiments [1]. For example, the HIV-1 Human Protein Interaction Database (HHPID) maintained by NIAID contains over 2500 functional connections between individual and human proteins observed over 25 years of research, approximately 30% of which are classified as physical binding interactions [2]. Another database, VirusMINT [3], contains a collection of literature-curated physical interactions for several viruses, the vast majority corresponding to HIV-1.

Several large-scale, systematic studies using the yeast two-hybrid methodology have recently been performed for several important human pathogens, including hepatitis C [4], Epstein-Barr [5], and influenza [6] viruses. Other approaches, such as those using Protein-fragment Complementation Assays (PCA) [7], protein arrays [8], or affinity tagging/purification combined with mass spectrometry (AP-MS) [9], which have been successfully used in other systems [10-13], have not been exploited to systematically interrogate host-pathogen physical relationships. We have, however, recently carried out the first systematic host-pathogen AP-MS study targeting HIV-1 using two different cell lines (HEK293 and Jurkat) (Jager et al., submitted), which will further increase the need for tools to visualize and integrate host-pathogen interaction datasets.

In addition to physical interaction studies, functionally important factors in HIV biology have also been identified by genetic or proteomic profiling screens. These studies do not necessarily identify physical binding partners for pathogenic proteins, but rather often implicate pathways or indirect "functional" associations. In 2008, three separate siRNA screens were published (Brass, Konig, and Zhou datasets) [14-16] that identified host genes required for efficient HIV infection. More recently, an additional RNAi screen was carried out using shRNAs in a potentially more physiologically relevant Jurkat cell line (Yeung dataset) [17]. RNAi studies in mammalian cells are also giving new insights into the host response to a number of other pathogenic organisms, including hepatitis C [18,19], influenza [20-23], West Nile [24], and Dengue fever viruses [25].

Similarly, several mass spectrometry-based studies examined protein expression levels in HIV-infected and uninfected cells. For example, Speijer and colleagues [26] used a 2D-DIGE approach in the human T-cell line PM1 where protein expression was measured following HIV infection. Another study examined protein abundance changes in a CD4 cell line 36 hours post-infection [27], whereas the most recent study reports on global protein level changes in primary CD4 cells isolated from five donors [28], profiling proteomic changes post infection in a time-dependent fashion.

**Figure 1:**
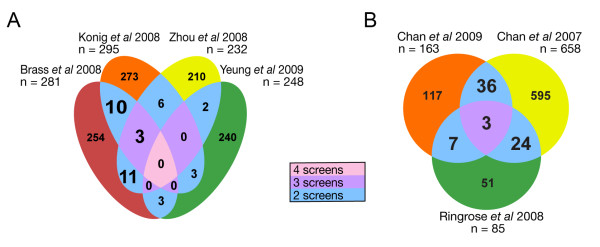
**Numerous host factors have been identified for HIV by small-scale and high-throughput experiments, with little overlap between the various sources**. (A) Venn diagram shows overlap from four HIV-based genetic screens [14-17]. Only three intersections show a significantly higher number of shared genes than expected, which are highlighted in large type. Ten genes are shared between the Brass and König datasets (p = 0.01), 11 between Brass and Zhou datasets (p = 0.0014), and three between Brass, König, and Zhou datasets (p = 5 × 10^-5^). None are shared between all four datasets. (B) Venn diagram shows a similar analysis for three HIV-dependent proteomic profiling screens [26-28]. Large type highlights statistically significant overlaps between the datasets (below 1 × 10^-4^).

At the most basic level, there exist two different types of data (physical vs. functional) and they both provide different insights into molecular mechanism. For example, genetic and proteomic profiling screens probing HIV-human interactions provide a wealth of data on genes and processes that contribute to pathogenesis but do not necessarily reflect direct physical connections. Conversely, methodologies that probe for physical interactions often miss crucial functional connections. Therefore, poor overlap is often seen when comparing datasets derived from these different, but complementary platforms. However, even a comparison of datasets collected using the same technology can reveal a very low overlap. For example, although the initial HIV RNAi screens each identified approximately 300 genes [14-16], there was a small (albeit statistically significant) overlap of three factors [29,30]. Several reasons contribute to this lack of concordance, including differences in the cell types (e.g., HeLa vs. HEK293T), the RNAi approaches and libraries used, as well as the phenotypic effects that were monitored. A comparison of all four genetic screens, which includes the most recent dataset derived from Jurkat cells using an shRNA library [17], finds no common factor between them (Figure 1A). In fact, only seven of 252 genes in this dataset are shared with even one of the other genetic screens (p = 0.654). Similarly, proteomic profiling datasets shared a low number of proteins (three) among all three datasets, although this is still statistically significant (p < 10^-5^, Figure 1B).

In cases where multiple types of data are available, it has been extremely illuminating to combine the diverse datasets to identify common pathways, processes, and complexes. For example, one recent study combined genetic and physical interaction data to identify new regulators of Wnt/β-Catenin signaling in mammalian cells [31]. Another study carried out a meta-analysis of several host-HIV-1 datasets, integrated with host protein-protein interaction databases, and reported significant overrepresented clusters within a network of host-pathogen and host-host interactions as important functional modules involved in virulence [29]. Another recent study identified key processes and host cellular subsystems impacted by HIV-1 infection by analyzing patterns of interactions in the HHPID, in combination with functional annotation and cross-referencing to global siRNA data [32].

In order to facilitate integration and exploration of the vast number of HIV-human interactions from different databases and data types, we have created a tool, termed GPS-Prot, with access to all major HIV-1 and human interaction databases as well as an option to overlay functional data (e.g. genetic interactions), which requires only very basic user input to produce an integrated network. To our knowledge this is the first tool to combine comprehensive HIV-1 and human physical/functional interaction data with a graphical viewer and web interface. Users can thus apply the GPS-Prot platform as a "global positioning system" to visualize any human-HIV-1 interaction in the context of its landscape of reported binding partners. We have also implemented a feature for users to securely upload and view their own datasets of interest. This software uses a unique graphical interface based on TouchGraph LLC's Navigator program, which has been used for social networking applications and which makes navigating and gathering information from large networks intuitive and rapid. We therefore suggest that GPS-Prot is ideal for a novice user to quickly and easily build human-HIV-1 interaction networks from the wealth of published information, or from a user's own dataset, and to expand the network around a particular protein of interest.

## Implementation

### Analysis of overlapping genes/proteins

Gene lists were obtained from four genetic screens [14-17] and three proteomic profiling studies [26-28] and converted to NCBI Entrez gene identifiers. A list of published and converted identifiers for all screens can be found in Additional file 1 (see Additional file 1: identifiers.xls). Statistical significance of gene/protein overlaps was calculated using frequency of overlap in size-matched, randomly generated datasets.

### Development of GPS-Prot

GPS-Prot is hosted on an Apache 2.0 web server and data retrieved from external databases resides in a MySQL relational database. Identifiers are mapped to Entrez GeneIDs. The logic tier is handled by PHP5 and the output of each database search is an XML file describing (1) individual proteins and (2) binary interactions. This file is passed to the network viewer, a version of TouchGraph Navigator (java applet) that is customized for our application. A spring-embedded layout is created within Navigator to view and navigate through the network, along with data tables containing information about the proteins and interactions. The Navigator applet performs well with up to 100,000 nodes and 200,000 edges, which is larger than any network that typical users will encounter. A connection to the server can be established within the applet allowing subsequent searches to be carried out by double-clicking on proteins in the network with the new interactions being added to the existing network.

Human PPIs are taken from six publicly available human interaction databases (downloaded June 2011; to be updated quarterly): HPRD [33] (Release 8), IntAct [34], MINT [35], BioGRID [36], DIP [37], and MIPS [38]. VirusMINT [3] (downloaded June 2011, to be updated quarterly) is used as the default HIV-human interaction database in GPS-Prot. Each interaction is linked to PubMed identifiers (PMID) and experimental descriptors and all protein identifiers are converted to Entrez gene nomenclature to facilitate identification of duplicate entries, which are consolidated for scoring purposes. The seven functional screens discussed here are also searched by default (1763 factors).

Additional optional databases currently include HIV-BIND (a subset of BIND containing HIV-human interactions) [39], the NIAID HIV-1 Human Database (HHPID) [40] from which many of the interactions in VirusMINT are derived, CORUM [41], and a published set of predicted HIV-human interactions (3372 interactions) [42].

To simplify searching and viewing, we do not separate viral proteins according to strains. All interactions imported from the various databases are mapped to the representative virus protein name.

To facilitate visualization of large networks, each physical interaction in the network is assigned a score. A high score indicates that an interaction has been reported in several independent publications, or perhaps only once, but with a high-confidence experimental technique (e.g. NMR or x-ray crystallography). The method is a modification of that used by the MINT database [35], which has been adapted for use across multiple databases, where curation standards and reported details of experiments vary (see Additional file 2; Additional_methods.doc). The optional database of CORUM complexes is treated as if all subunits interact and scored as 1.0 so that they are retained in the networks at any scoring threshold. The output of a search is an XML file, viewed using a customized applet for PPIs that appears in the GPS-Prot Navigator window (TouchGraph LLC, New York, NY).

User upload of data (up to nine datasets) is permitted after creating an account at the GPS-Prot website. Uploaded data can be of two types: physical interactions or genetic/functional interactions. Physical interactions should be formatted as a two-column list of interacting proteins (Uniprot or Entrez identifiers, tab delimited; e.g., .txt file from Microsoft Excel). Genetic/functional interactions should be formatted as a single column list of Uniprot or Entrez identifiers. At present, only HIV or human proteins can be uploaded.

### Analysis of overlapping complexes/functional modules

Datasets were analyzed in terms of subunits of complexes or functional modules defined by CORUM [41]. Because CORUM includes subunits interacting with multiple complexes or subcomplexes, we created an all-against-all binary matrix of protein interactions to assign subunits to unique complexes or functional modules. This was necessary to assign one complex and its subunits to one intersection of the datasets. Hierarchical clustering was carried out on the matrix using Cluster 3.0 and a branch length threshold of 1.6 was used to select clusters from the dendrogram, which we defined as our set of complexes, after some manual refinement (see Additional file 3: Corum_compl.xls). In total, the set consists of 222 complexes, containing 1600 subunits (see Additional file 3: Corum_compl.xls). Genes/proteins from the datasets were assigned to complexes/functional modules and the overlaps of complexes between the different datasets calculated. Statistical significance of the number of subunits overlapping was calculated using frequency observed in size-matched, randomly generated datasets. In addition, the significance of the number of subunits identified in each complex was calculated using the hypergeometric distribution function in Microsoft Excel, (see Additional files 4 and 5: RNAi_compl.xls and Prot_compl.xls).

### Identification and verification of Vif complexes

Vif-binding proteins were identified by affinity tagging/purification combined with mass spectrometry analysis (Jager et al., submitted). To investigate further the novel interaction with Huwe1, we performed immunoprecipitations and Western blotting as follows: Plasmids that express Vif, Vpr, or Nef were constructed by inserting cDNA-derived genes into a pcDNA3 vector containing C-terminal tandem 2xStrep/3xFLAG tags, and 293 cells were transfected using calcium phosphate. Cells were harvested two days post-transfection and lysed and immunoprecipitated with anti-FLAG M2 affinity resin (Sigma) according to manufacturer instructions. Proteins eluted with 3xFLAG peptide were analyzed by Western blot using anti-Cul5, anti-UPF1 and anti-Elongin B (TCEB2) (Santa Cruz), anti-FLAG (Sigma), or anti-Huwe1 (Bethyl Laboratories) antibodies. Western blots were developed using ECL Plus Western Blotting Detection System (GE Healthcare).

## Results

### Generation of HIV-1-human networks using GPS-Prot

**Figure 2:**
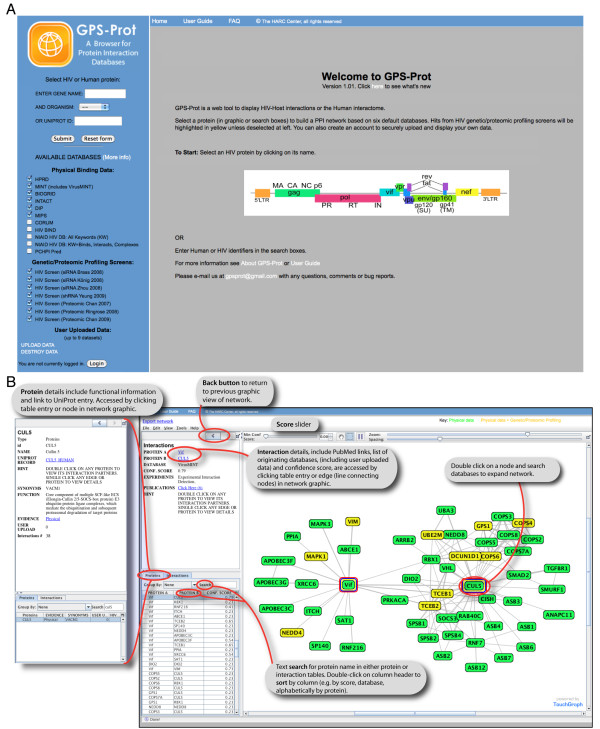
**GPS-Prot: a web-based platform for visualizing diverse HIV-host data**. (A) GPS-Prot homepage. Searches are initiated by selecting databases and an HIV or host protein. (B) A Touchgraph Navigator window is launched to display results of a search, which contains the protein interaction network. Single clicking any interaction ("edge", or gray line connecting proteins) provides the evidence from the literature for that interaction in the left-hand panel. Clicking on any protein in the diagram ("node") pulls up details for that protein (e.g. panel labeled CUL5). There is also a searchable table that can be sorted by score, database or experiment. A new network can be created by double clicking any protein (node), thus, it is possible to "walk through" the entire HIV-human or human-human interactome.

The GPS-Prot platform, found at http://www.gpsprot.org, allows users to initiate searches either by selecting an HIV protein from a graphic of the viral genome or by entering an HIV or human gene identifier in the search box (Figure 2A). A network is then generated and visualized (Figure 2B) using data from several publicly-available protein interaction databases, including VirusMINT [3] for HIV-host interactions, and HPRD [33], IntAct [34], MINT [43], BioGRID [36], DIP [37] and MIPS [38] for interactions between human proteins. There are also additional databases that can be selected.

The GPS-Prot databases selected on the homepage can also be searched from within the Navigator window by double clicking any node. Thus, it is possible to visualize not only the HIV-host interactions but also to explore second-shell (or third-shell, etc.) host-host interactions in an intuitive manner. Figure 2B shows a network with all human binding partners to the HIV Vif protein. In this case, after the initial network of Vif binders was built, the binding partners of CUL5, a factor hijacked by Vif [44], were added into the network by double clicking the CUL5 node (Figure 2B, right-most network).

Two text panels are located to the left of the network window. The top panel toggles to display two types of information depending on what is selected in the network: details about any protein (node) or any interaction (edge) (e.g. panels headed "CUL5" and "Interactions", respectively) (Figure 2B). Single clicking any node or edge toggles between the windows and includes information about the originating database(s) for the PPI (protein-protein interaction), experiment type, links to publications, functional information, and Uniprot entries.

Two tabs in the bottom left panel allow users to toggle between two tables that provide further details about the network. The "Protein" tab lists all proteins or nodes while the "Interactions" tab lists all interactions or edges. By default, a limited amount of information is included for each protein or interaction, which can be expanded to include additional parameters. For example, a useful "keywords" field can be added to the interactions table when using the NIAID HHPID database, and then interactions can be sorted by clicking on the column headers. Groups of table entries can be selected (e.g. all having the same keyword), causing them to be highlighted in the network panel. The search box can be used to find any particular protein in the loaded network.

We have assigned rough "confidence scores" to each pair-wise interaction based on the number of independent publications and experimental methods (see Implementation), similar in concept to the scoring used by the MINT database [43]. However, the scores used by GPS-Prot are not meant to evaluate the validity of interactions in any absolute way, but rather to allow users to dynamically change the number of viewed nodes by adjusting a confidence score slider in the network panel (Figure 2B), thereby acting as a filter to help visualize large networks with many nodes. The edge line widths in the network panel are also displayed in proportion to their scores and future quantitative information about HIV-human interactions can be incorporated later. For example, we have devised the MiST (mass spectrometry interaction statistics) score to quantitatively report on interactions derived from systematic AP-MS studies (Jager et al., submitted) and these values can be effectively incorporated into GPS-Prot.

The Navigator window also includes other features to help simplify visualization, such as zoom and spacing sliders (Figure 2B) and the ability to resize the information and network panels by dragging borders. Network images can be exported using a "Save Image" option under the File pulldown menu. Data can also be exported in the form of a tab-delimited file by using the "Export network" link in the Navigator window.

### Overlay of physical and functional interaction networks

One challenge in handling large-scale genomic datasets is the difficulty in integrating different data types, a task accomplished in GPS-Prot by allowing users to view data from functional screens in the context of PPI networks. By default, GPS-Prot includes seven genetic and proteomic profiling screens carried out in the context of HIV-1 infection [14-17,26-28], which are overlaid on the physical binding networks (Figure 2). Operationally, the physical interaction network is first built from the PPI databases (green nodes) and then interactors identified by the genetic or proteomic screens are highlighted in yellow, with links to publications in the information panel. Including functional data in a GPS-Prot search can highlight relevant clusters in a network. For example, the well-established complex of Vif with TCEB1 (Elongin C), TCEB2 (Elongin B) (which forms a larger complex with the Ring Box protein RBX1, and CUL5) [44], is easily noted in Figure 2B, as the Elongin subunits are highlighted in yellow based on RNAi and proteomic profiling screens. The importance of this complex during the HIV life cycle is well appreciated, as Vif targets APOBEC3G for degradation during the course of infection [44].

### Use of CORUM to identify complexes involved in HIV function

Another important feature of GPS-Prot is the ability to group subunits of complexes together by including data from the CORUM database [41], a collection of manually curated mammalian protein complexes. To date, there are several examples of HIV proteins interacting with well-characterized human complexes. For example, Tat interacts with CCNT1/CDK9, components of the elongation factor pTEFb, along with the chromatin regulators, AFF4, ENL, ELL, and AF9 [45,46], a complex important for transcriptional activation, and as previously mentioned, Vif hijacks a multi-subunit ubiquitin ligase complex containing Cul5, thus targeting APOBEC3G to the proteasome for degradation [44]. Analyzing and visualizing datasets in terms of complexes can increase agreement between different functional screens, which often have little overlap at the individual gene or protein level (Figure 1; [29]).

**Figure 3:**
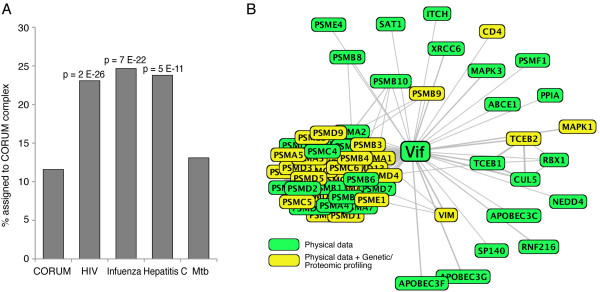
**Viral RNAi screens are enriched for host factors that are subunits of human complexes**. (A) All viral RNAi screens identify significantly more human complex subunits identified than expected (HIV 23%, influenza 25%, and hepatitis C 24%), compared to the number of proteins in the human genome assigned to complexes by CORUM (12%). P values shown are based on the hypergeometric distribution. We find no strong enrichment of protein complexes in a screen of Mtb host factors (13%). (B) Network of Vif interactors from GPS-Prot using the optional NIAID HIV-1-human interactions database, instead of VirusMINT. Including CORUM as a database brings complex subunits closer together in the network, for example the cluster of proteasome complex subunits shown to the lower left (e.g. PSMA, PSMB, PSMC, etc).

We used the CORUM database to identify statistically significant overlaps between genetic and proteomic screens. Initially, we found that the four HIV RNAi screens [14-17] are enriched for proteins that are part of protein complexes (Figure 3A), as annotated by CORUM. This trend was also observed for other small viruses for which RNAi data is available (Figure 3A), including hepatitis C [18,19] and influenza [20,22,23]. To see how these trends compared to genetic data derived from a bacterial pathogen, we analyzed a recent RNAi screen that assessed effects of Mycobacterium tuberculosis (Mtb) infection [47]. In this case we found no strong enrichment for subunits of protein complexes within the dataset (Figure 3A, p = 0.05). This was not due to an abundance of weakly expressing genes in the Mtb screen that could cause under-representation in the CORUM database (Additional file 6; Figure S1.doc). The observation that HIV and other viruses appear to target larger molecular machines compared to Mtb is consistent with the hypothesis that its significantly smaller genome (15 proteins vs. ~4000 in Mtb) requires that it needs to physically hijack a greater proportion of the host machinery.

**Figure 4:**
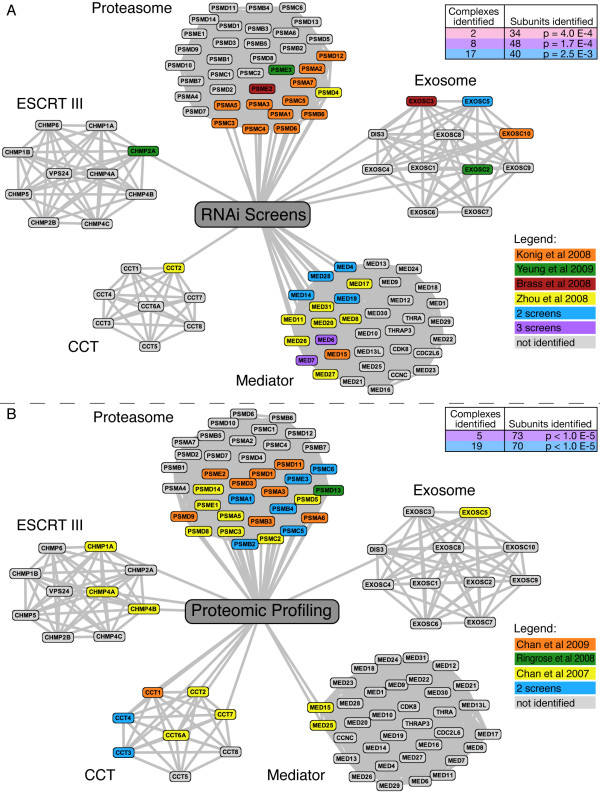
**Five complexes implicated in HIV pathogenesis by analysis with CORUM**. (A) Network analysis of RNAi datasets. Gray nodes are subunits present in the complex according to the CORUM database. Colored subunits (nodes) were reported in one or more of the genetic screens. Based on the hypergeometric distribution, we find significantly more subunits of the proteasome (p = 4.2 × 10^-9^), Mediator (p = 1.1 × 10^-9^), and the exosome (p = 2.1 × 10^-3^) than expected. Subunits of ESCRT III and CCT complexes are not significantly enriched. The table shows the number of complexes and subunits identified by two, three or four RNAi screens. As with genetic screens, there is greater overlap between datasets when analyzed in terms of subunits of complexes as opposed to isolated proteins. (B) Network analysis of proteomic profiling datasets. The same complexes are shown as in panel A, with subunits highlighted as they occur in different datasets. Mediator and exosome complexes are not covered more than expected, but significantly more subunits than expected are found for ESCRT III (p = 8.4 × 10^-3^) and CCT complexes (p = 2.0 × 10^-7^). The proteasome is the only complex where more subunits than expected are identified by both genetic and proteomic profiling screens (p = 7.0 × 10^-23^).

Our analysis also shows that HIV-1 RNAi datasets have a greater intersection when they are analyzed in terms of multi-subunit complexes rather than as individual factors. The tables in Figure 4 show the number of subunits from the same complex identified in the RNAi (Figure 4A) and proteomic screens (Figure 4B). For example, both the spliceosome and proteasome were identified in all four genetic screens and included 34 subunits (p = 4.0 × 10^-4^) of these two complexes (20 and 14 subunits, respectively) (p = 2.9 × 10^-6^, p = 4.8 × 10^-9 ^respectively) (Additional file 4:RNAi_compl.xls). In all, 48 proteins (p = 1.7 × 10^-4^) belonging to eight separate complexes and 40 proteins (p = 2.5 × 10^-3^) belonging to 17 separate complexes were identified in three and two screens, respectively (Additional file 4: RNAi_compl.xls). Collectively, there were 1014 proteins identified in all four RNAi screens, of which 122 are found in at least two screens when analyzed in the context of a protein complex (p < 10^-5^).

A similar concordance is found in the proteomic profiling datasets when analyzed in the context of protein complexes (Figure 4B, Additional file 5:Prot_compl.xls). In total, 120 complexes are implicated in HIV function by all seven datasets (Additional files 4 and 5: RNAi_compl.xls and Prot_compl.xls). Some complexes were identified by both technologies, including the proteasome (Figure 4A and 4B), while others were only significantly enriched in one, such as ESCRT III in the proteomic profiling screens. Overall, 38 complexes are identified by both genetic and proteomic profiling, 48 by genetic screening alone, and 34 by proteomic profiling alone.

To confirm this analysis, we sought to verify one of these identified complexes experimentally. This was accomplished by knockdown of a set of mediator subunits that were not identified in any screen as host factors (gray subunits in Figure 4). We found that RNAi targeted to one of these, MED30, strongly inhibited early-stage HIV replication without inducing toxicity (Additional file 7; Figure S2.doc). MED30 is contained within the head module of Mediator, one of four functionally distinct sub-complexes [48], and is required for promoter recognition [49] and assembly/stabilization of transcription pre-initiation complexes [50,51]. Interestingly, RNAi knockdown of 8 out of 11 (p = 0.007) head module factors (including MED30) affect replication while no protein in the Cdk8 module was identified in any of the RNAi screens (see Additional file 4: RNAi_compl.xls).

Based on this analysis, we conclude that analyzing the genetic data in the context of complexes is useful for identifying statistically significant factors affecting HIV function. Allowing users to optionally select CORUM in GPS-Prot permits a similar analysis, albeit at a visual level, by highlighting complexes with different subunits that have been identified in different screens. We have found that including data from the CORUM database can increase the visual overlap between different genetic and proteomic screens and allow users to disentangle biochemical complexes from broader biological processes. Figure 3B shows the visual advantage of including CORUM in a search; in this case, using it in conjunction with the NIAID HIV-1-human interactions database. GPS-Prot presumes an edge between all members of a complex, bringing members in the network into a very dense cluster of nodes. As shown in Figure 4, different subunits of the proteasome are identified in all seven HIV functional screens. The proteasome is much more clearly identified as a complex, in GPS-Prot when CORUM data is included.

The approach of combining information from different screens, particularly those utilizing different technologies, is effective, in part, because many screens do not reach saturation. There can also be a high false negative rate (e.g. known binders of HIV proteins, such as Cyclin T1, are not found in some screens) or false positive rate, due to off target effects and variable expression of host factors in different cell lines. Analyses in the context of complexes compensates to some extent for these limitations by identifying overlaps between datasets, especially when saturation is not reached.

### Upload of user-generated data

According to the HHPID database, numerous host factors (up to several hundred) may interact with any given HIV-1 protein. In addition, RNAi screens alone have added more than 800 unique host factors to the current datasets. The continuing issue when obtaining new datasets is to distinguish between relevant hits and noise, which can be aided, as we have shown, by combining multiple datasets and/or analyzing the data in the context of protein complexes. To address this need, GPS-Prot allows users to create an account and upload up to nine in-house datasets to be included in the interaction networks. The set can describe physical interactions, consisting of a list of binary interacting proteins, or simply a list of genes/proteins such as that generated by RNAi or proteomic profiling screens (see Implementation for details).

**Figure 5:**
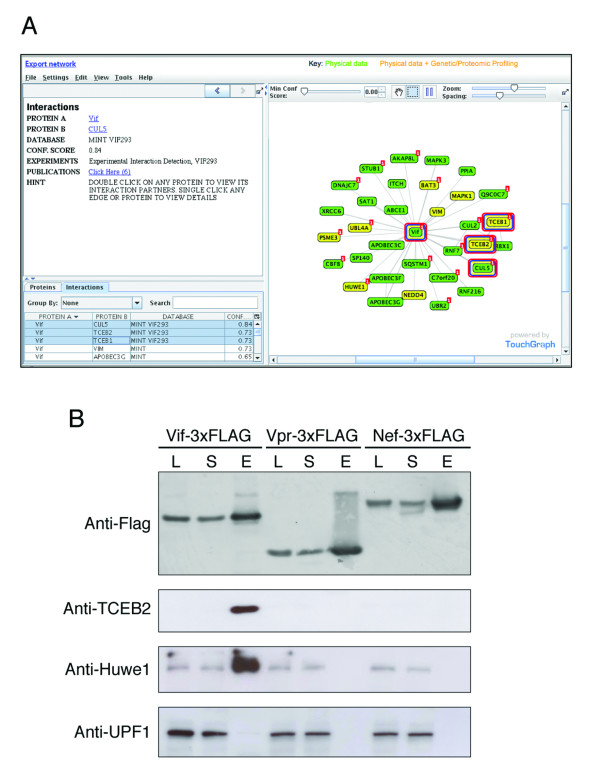
**User-generated data can be uploaded and viewed in the context of complete PPI networks from public databases**. (A) Vif network from GPS-Prot, including an uploaded dataset from AP-MS experiments (red-tagged nodes). Huwe1 is among several proteins in the uploaded dataset (Jager et al., submitted) that are not found in other databases (e.g., not present in Figure 2B), and were also previously identified by genetic/proteomic screens. (B) HIV Vif interacts with endogenous HUWE1 in 293 cells. 3xFLAG-tagged Vif, Vpr, and Nef were immunoprecipitated with anti-FLAG agarose beads. Lysates (L), remaining supernatant (S) and eluates (E) were analyzed by SDS-PAGE and Western blotting with antibodies as indicated. The same band is identified in the Vif pulldown by antibodies against the known CUL5 E3 ligase complex, anti-CUL5 (not shown) and anti-ELOB (TCEB2) as well as anti-Huwe1 antibodies, but not by the control anti-UPF1 antibody.

We used this feature to analyze a partial dataset from our ongoing project to determine a comprehensive human-HIV-1 interaction map using AP-MS [52] (Jager et al., submitted). We obtained preliminary interaction data for Vif by transiently expressing and purifying a C-terminally 3xFLAG tagged version from HEK293 cells and analyzed the associated proteins by mass spectrometry. We then uploaded these data into GPS-Prot, to view in the context of previously reported Vif binders (Figure 5A; uploaded data are marked with red tags). The most well-characterized Vif partners, TCEB1 (Elongin C), TCEB2 (Elongin B), and CUL5 (circled in red and highlighted in the lower left table), were present in the AP-MS dataset and two of these (TCEB1 and TCEB2) were also found in RNAi and/or proteomic screens (yellow nodes). Interestingly, of the four remaining proteins observed both by AP-MS and in the screens (yellow and red-tagged), three of these, PSME3 (a proteasome subunit), HUWE1 (an E3 ligase), and UBL4A (a ubiquitin-like protein), have functions that may relate to the role of Vif in ubiquitin-tagging substrates for proteasomal degradation. Because Huwe1 acts during the late stages of HIV infection [14] when Vif is believed to function, we retested the Vif-Huwe1 interaction by immunoprecipitation (IP)-Western blotting using an antibody against Huwe1 and indeed observed strong and specific binding (Figure 5B). It will be of great interest to determine whether Vif itself is targeted for ubiquitination by Huwe1 or whether Huwe1 might be a second ubiquitin ligase recruited by Vif to tag APOBEC3G or other as-yet-unidentified targets for degradation.

### Comparison with other platforms

There are a number of tools for visually exploring biological networks, such as PINA [53], STRING [54], Cytoscape [55], and others (reviewed in [56]). Some standalone databases are also integrated with viewers, such as the MINT database [57]. Others are linked to external viewers such as Osprey [58] for BioGRID database interactions or the Cytoscape plugin MiSink for DIP interactions [59]. Alternatively, sites like STRING and APID/APID2NET have plug-ins for Cytoscape [60] and integrate interactome data from multiple PPI databases.

Many of the existing network analysis platforms, however, do not include HIV-host interactions, or virus-host interactions in general, and also require varying degrees of expert knowledge to produce and navigate networks. Thus, there is a need to integrate and synthesize the abundant HIV-host physical and genetic interaction information (or more generally host-pathogen information) from public repositories. PIG [61] and VirusMINT [3] have taken steps in this direction by creating databases that contain a substantial number of physical HIV interactions, along with other physical virus-host interactions. CAPIH is a tool that provides a web interface for accessing physical host-HIV interactions [62] in the context of comparative genome analysis and provides information about the differences in sequences between interacting proteins of model organisms (chimpanzee, rhesus macaque, and mouse). Also, a web version of JNets [63] allows users to view a global network representation of the HHPID HIV-host interactions and explore that network using the underlying annotations, such as Gene Ontology (GO) annotation or HHPID keywords.

Aside from the issue of integrating physical and genetic virus-host data, it has been noted that some biological network tools utilize generic graph drawing tools that are not necessarily intuitive to most biologists [56]. We took an alternative approach of harnessing a commercial viewer (TouchGraph Navigator), which has been developed for non-scientific applications including social network analysis, and modifying it in collaboration with its designers for our scientific application.

GPS-Prot also allows users to include information about complexes through inclusion of data from the CORUM database. Our results suggest this approach may be particularly suited to viruses or other pathogens that rely extensively on multi-subunit host machinery, as indicated by our preliminary comparison with the bacterial pathogen Mtb. However the vast majority of data available are from viral pathogens and more studies of microbe pathogens are required to definitively tease apart the differences.

## Conclusions

As high-throughput technologies identify more host factors that physically associate with viral factors, it is vital to integrate this information with other, diverse types of data, such as genetic and proteomic profiling, and to provide tools to visualize them in intuitive ways. GPS-Prot provides such a tool by aggregating several major databases for physical virus-host and host-host PPIs and overlaying HIV-1 genetic/proteomic profiling data, in addition to allowing upload of new user-generated data.

A next goal is to extend the GPS-Prot infrastructure to other pathogens, particularly viruses. Currently very few have datasets as large as HIV-1, particularly with regard to the physical interactome of each viral protein. We have collected physical interaction datasets derived from AP-MS studies for HIV-1 in HEK293 and Jurkat cells that will be included in the GPS-Prot set of databases (Jager et al., submitted). Finally, we also intend to expand these analyses to other pathogens in the near future.

## Availability and Requirements

GPS-Prot is freely available to all users with Java-enabled web browsers (best viewed with Safari and Firefox) at http://www.gpsprot.org. GPS-Prot was coded using XHTML, CSS, PHP, XML, Java, MySQL and jQuery.

## List of Abbreviations

PPI: Protein-protein interaction

## Competing interests

The authors declare that they have no competing interests.

## Authors' contributions

MEF, MJB, ADF and NJK designed the approach, analyzed data and wrote the manuscript. MEF, CM, SJ, LP, DK, KR, SKC, CSC collected results and analyzed data. All authors read and approved the final manuscript. MEF and MJB designed and implemented GPS-Prot website. AS designed and implemented customized Navigator applet.

## Supplementary Material

Additional file 1 **Published and converted identifiers for all seven HIV screens**. Click here for file

Additional file 2 Click here for file

Additional file 3 **Dataset of 222 human complexes derived from CORUM by clustering and details of manual refinement of complexes**. Click here for file

Additional file 4 **Complexes and subunits identified by RNAi studies**. Click here for file

Additional file 5 **Complexes and subunits identified by proteomic profiling studies**. Click here for file

Additional file 6 **Comparison of broad expression level of Mtb and HIV screens**. Click here for file

Additional file 7 **RNAi-mediated depletion of MED30 blocks early steps of replication of a VSV-G pseudotyped HIV luciferase virus**. Click here for file

## References

[B1] Dyer MD Murali TM Sobral BW The landscape of human proteins interacting with viruses and other pathogens PLoS Pathog 2008 4 2 e32 10.1371/journal.ppat.0040032 18282095 PMC2242834

[B2] Fu W Sanders-Beer BE Katz KS Maglott DR Pruitt KD Ptak RG Human immunodeficiency virus type 1, human protein interaction database at NCBI Nucleic Acids Res 2009 37 Database D417 422 18927109 10.1093/nar/gkn708 PMC2686594

[B3] Chatr-aryamontri A Ceol A Peluso D Nardozza A Panni S Sacco F Tinti M Smolyar A Castagnoli L Vidal M Cusick ME Cesareni G VirusMINT: a viral protein interaction database Nucleic Acids Res 2009 37 Database D669 673 18974184 10.1093/nar/gkn739 PMC2686573

[B4] de Chassey B Navratil V Tafforeau L Hiet MS Aublin-Gex A Agaugué S Meiffren G Pradezynski F Faria BF Chantier T Le Breton M Pellet J Davoust N Mangeot PE Chaboud A Penin F Jacob Y Vidalain PO Vidal M André P Rabourdin-Combe C Lotteau V Hepatitis C virus infection protein network Mol Syst Biol 2008 4 230 18985028 10.1038/msb.2008.66 PMC2600670

[B5] Calderwood MA Venkatesan K Xing L Chase MR Vazquez A Holthaus AM Ewence AE Li N Hirozane-Kishikawa T Hill DE Vidal M Kieff E Johannsen E Epstein-Barr virus and virus human protein interaction maps Proc Natl Acad Sci USA 2007 104 18 7606 7611 10.1073/pnas.0702332104 17446270 PMC1863443

[B6] Shapira SD Gat-Viks I Shum BOV Dricot A de Grace MM Wu L Gupta PB Hao T Silver SJ Root DE Hill DE Regev A Hacohen N A physical and regulatory map of host-influenza interactions reveals pathways in H1N1 infection Cell 2009 139 7 1255 1267 10.1016/j.cell.2009.12.018 20064372 PMC2892837

[B7] Tarassov K Messier V Landry CR Radinovic S Serna Molina MM Shames I Malitskaya Y Vogel J Bussey H Michnick SW An in vivo map of the yeast protein interactome Science 2008 320 5882 1465 1470 10.1126/science.1153878 18467557

[B8] MacBeath G Schreiber SL Printing Proteins as Microarrays for High-Throughput Function Determination Science 2000 289 5485 1760 10976071 10.1126/science.289.5485.1760

[B9] Puig O Caspary F Rigaut G Rutz B Bouveret E Bragado-Nilsson E Wilm M Séraphin B The tandem affinity purification (TAP) method: a general procedure of protein complex purification Methods 2001 24 3 218 229 10.1006/meth.2001.1183 11403571

[B10] Gavin A-CC Aloy P Grandi P Krause R Boesche M Marzioch M Rau C Jensen LJ Bastuck S Dümpelfeld B Edelmann A Heurtier M-AA Hoffman V Hoefert C Klein K Hudak M Michon A-MM Schelder M Schirle M Remor M Rudi T Hooper S Bauer A Bouwmeester T Casari G Drewes G Neubauer G Rick JM Kuster B Bork P Proteome survey reveals modularity of the yeast cell machinery Nature 2006 440 7084 631 636 10.1038/nature04532 16429126

[B11] Krogan NJ Cagney G Yu H Zhong G Guo X Ignatchenko A Li J Pu S Datta N Tikuisis AP Punna T Peregrín-Alvarez JM Shales M Zhang X Davey M Robinson MD Paccanaro A Bray JE Sheung A Beattie B Richards DP Canadien V Lalev A Mena F Wong P Starostine A Canete MM Vlasblom J Wu S Orsi C Global landscape of protein complexes in the yeast Saccharomyces cerevisiae Nature 2006 440 7084 637 643 10.1038/nature04670 16554755

[B12] Sowa ME Bennett EJ Gygi SP Harper JW Defining the human deubiquitinating enzyme interaction landscape Cell 2009 138 2 389 403 10.1016/j.cell.2009.04.042 19615732 PMC2716422

[B13] Behrends C Sowa ME Gygi SP Harper JW Network organization of the human autophagy system Nature 2010 466 7302 68 76 10.1038/nature09204 20562859 PMC2901998

[B14] Brass AL Dykxhoorn DM Benita Y Yan N Engelman A Xavier RJ Lieberman J Elledge SJ Identification of host proteins required for HIV infection through a functional genomic screen Science 2008 319 5865 921 926 10.1126/science.1152725 18187620

[B15] König R Zhou Y Elleder D Diamond TL Bonamy GMC Irelan JT Chiang C-YY Tu BP De Jesus PD Lilley CE Seidel S Opaluch AM Caldwell JS Weitzman MD Kuhen KL Bandyopadhyay S Ideker T Orth AP Miraglia LJ Bushman FD Young JA Chanda SK Global analysis of host-pathogen interactions that regulate early-stage HIV-1 replication Cell 2008 135 1 49 60 10.1016/j.cell.2008.07.032 18854154 PMC2628946

[B16] Zhou H Xu M Huang Q Gates AT Zhang XD Castle JC Stec E Ferrer M Strulovici B Hazuda DJ Espeseth AS Genome-scale RNAi screen for host factors required for HIV replication Cell Host Microbe 2008 4 5 495 504 10.1016/j.chom.2008.10.004 18976975

[B17] Yeung ML Houzet L Yedavalli VSRK Jeang K-TT A genome-wide short hairpin RNA screening of jurkat T-cells for human proteins contributing to productive HIV-1 replication J Biol Chem 2009 284 29 19463 19473 10.1074/jbc.M109.010033 19460752 PMC2740572

[B18] Li Q Brass AL Ng A Hu Z Xavier RJ Liang TJ Elledge SJ A genome-wide genetic screen for host factors required for hepatitis C virus propagation Proc Natl Acad Sci USA 2009 106 38 16410 16415 10.1073/pnas.0907439106 19717417 PMC2752535

[B19] Tai AW Benita Y Peng LF Kim S-SS Sakamoto N Xavier RJ Chung RT A functional genomic screen identifies cellular cofactors of hepatitis C virus replication Cell Host Microbe 2009 5 3 298 307 10.1016/j.chom.2009.02.001 19286138 PMC2756022

[B20] Brass AL Huang I-CC Benita Y John SP Krishnan MN Feeley EM Ryan BJ Weyer JL van der Weyden L Fikrig E Adams DJ Xavier RJ Farzan M Elledge SJ The IFITM proteins mediate cellular resistance to influenza A H1N1 virus, West Nile virus, and dengue virus Cell 2009 139 7 1243 1254 10.1016/j.cell.2009.12.017 20064371 PMC2824905

[B21] Hao L Sakurai A Watanabe T Sorensen E Nidom CA Newton MA Ahlquist P Kawaoka Y Drosophila RNAi screen identifies host genes important for influenza virus replication Nature 2008 454 7206 890 893 10.1038/nature07151 18615016 PMC2574945

[B22] Karlas A Machuy N Shin Y Pleissner K-PP Artarini A Heuer D Becker D Khalil H Ogilvie LA Hess S Mäurer AP Müller E Wolff T Rudel T Meyer TF Genome-wide RNAi screen identifies human host factors crucial for influenza virus replication Nature 2010 463 7282 818 822 10.1038/nature08760 20081832

[B23] König R Stertz S Zhou Y Inoue A Hoffmann H-HH Bhattacharyya S Alamares JG Tscherne DM Ortigoza MB Liang Y Gao Q Andrews SE Bandyopadhyay S De Jesus P Tu BP Pache L Shih C Orth A Bonamy G Miraglia L Ideker T García-Sastre A Young JAT Palese P Shaw ML Chanda SK Human host factors required for influenza virus replication Nature 2010 463 7282 813 817 10.1038/nature08699 20027183 PMC2862546

[B24] Krishnan MN Ng A Sukumaran B Gilfoy FD Uchil PD Sultana H Brass AL Adametz R Tsui M Qian F Montgomery RR Lev S Mason PW Koski RA Elledge SJ Xavier RJ Agaisse H Fikrig E RNA interference screen for human genes associated with West Nile virus infection Nature 2008 455 7210 242 245 10.1038/nature07207 18690214 PMC3136529

[B25] Sessions OM Barrows NJ Souza-Neto JA Robinson TJ Hershey CL Rodgers MA Ramirez JL Dimopoulos G Yang PL Pearson JL Garcia-Blanco MA Discovery of insect and human dengue virus host factors Nature 2009 458 7241 1047 1050 10.1038/nature07967 19396146 PMC3462662

[B26] Ringrose JH Jeeninga RE Berkhout B Speijer D Proteomic studies reveal coordinated changes in T-cell expression patterns upon infection with human immunodeficiency virus type 1 J Virol 2008 82 9 4320 4330 10.1128/JVI.01819-07 18287243 PMC2293043

[B27] Chan EY Qian W-JJ Diamond DL Liu T Gritsenko MA Monroe ME Camp DG Smith RD Katze MG Quantitative analysis of human immunodeficiency virus type 1-infected CD4+ cell proteome: dysregulated cell cycle progression and nuclear transport coincide with robust virus production J Virol 2007 81 14 7571 7583 10.1128/JVI.00288-07 17494070 PMC1933372

[B28] Chan EY Sutton JN Jacobs JM Bondarenko A Smith RD Katze MG Dynamic host energetics and cytoskeletal proteomes in human immunodeficiency virus type 1-infected human primary CD4 cells: analysis by multiplexed label-free mass spectrometry J Virol 2009 83 18 9283 9295 10.1128/JVI.00814-09 19587052 PMC2738255

[B29] Bushman FD Malani N Fernandes J D'Orso I Cagney G Diamond TL Zhou H Hazuda DJ Espeseth AS Konig R Bandyopadhyay S Ideker T Goff SP Krogan NJ Frankel AD Young JA Chanda SK Host cell factors in HIV replication: meta-analysis of genome-wide studies PLoS Pathog 2009 5 5 e1000437 10.1371/journal.ppat.1000437 19478882 PMC2682202

[B30] Goff SP Knockdown screens to knockout HIV-1 Cell 2008 135 3 417 420 10.1016/j.cell.2008.10.007 18984154 PMC3598618

[B31] Major MB Roberts BS Berndt JD Marine S Anastas J Chung N Ferrer M Yi X Stoick-Cooper CL von Haller PD Kategaya L Chien A Angers S MacCoss M Cleary MA Arthur WT Moon RT New regulators of Wnt/beta-catenin signaling revealed by integrative molecular screening Sci Signal 2008 1 45 ra12 10.1126/scisignal.2000037 19001663

[B32] Macpherson J Pinney JW Robertson DL Patterns of HIV-1 protein interaction identify perturbed host-cellular subsystems PLoS Comput Biol 2010 6 7 e1000863 10.1371/journal.pcbi.1000863 20686668 PMC2912648

[B33] Keshava Prasad TS Goel R Kandasamy K Keerthikumar S Kumar S Mathivanan S Telikicherla D Raju R Shafreen B Venugopal A Balakrishnan L Marimuthu A Banerjee S Somanathan DS Sebastian A Rani S Ray S Harrys Kishore CJ Kanth S Ahmed M Kashyap MK Mohmood R Ramachandra YL Krishna V Rahiman BA Mohan S Ranganathan P Ramabadran S Chaerkady R Pandey A Human Protein Reference Database-2009 update Nucleic Acids Res 2009 37 Database D767 772 18988627 10.1093/nar/gkn892 PMC2686490

[B34] Kerrien S Alam-Faruque Y Aranda B Bancarz I Bridge A Derow C Dimmer E Feuermann M Friedrichsen A Huntley R Kohler C Khadake J Leroy C Liban A Lieftink C Montecchi-Palazzi L Orchard S Risse J Robbe K Roechert B Thorneycroft D Zhang Y Apweiler R Hermjakob H IntAct-open source resource for molecular interaction data Nucleic Acids Res 2007 35 Database D561 565 17145710 10.1093/nar/gkl958 PMC1751531

[B35] Chatr-aryamontri A Ceol A Palazzi LM Nardelli G Schneider MV Castagnoli L Cesareni G MINT: the Molecular INTeraction database Nucleic Acids Res 2007 35 Database D572 574 10.1093/nar/gkl950 PMC1751541 17135203

[B36] Breitkreutz B-JJ Stark C Reguly T Boucher L Breitkreutz A Livstone M Oughtred R Lackner DH Bähler J Wood V Dolinski K Tyers M The BioGRID Interaction Database: 2008 update Nucleic Acids Res 2008 36 Database D637 640 18000002 10.1093/nar/gkm1001 PMC2238873

[B37] Xenarios I Salwínski L Duan XJ Higney P Kim S-MM Eisenberg D DIP, the Database of Interacting Proteins: a research tool for studying cellular networks of protein interactions Nucleic Acids Res 2002 30 1 303 305 10.1093/nar/30.1.303 11752321 PMC99070

[B38] Mewes HW Frishman D Mayer KFX Münsterkötter M Noubibou O Pagel P Rattei T Oesterheld M Ruepp A Stümpflen V MIPS: analysis and annotation of proteins from whole genomes in 2005 Nucleic Acids Res 2006 34 Database D169 172 10.1093/nar/gkj148 PMC1347510 16381839

[B39] Alfarano C Andrade CE Anthony K Bahroos N Bajec M Bantoft K Betel D Bobechko B Boutilier K Burgess E Buzadzija K Cavero R D'Abreo C Donaldson I Dorairajoo D Dumontier MJ Dumontier MR Earles V Farrall R Feldman H Garderman E Gong Y Gonzaga R Grytsan V Gryz E Gu V Haldorsen E Halupa A Haw R Hrvojic A The Biomolecular Interaction Network Database and related tools 2005 update Nucleic Acids Res 2005 33 Database D418 424 15608229 10.1093/nar/gki051 PMC540005

[B40] Ptak RG Fu W Sanders-Beer BE Dickerson JE Pinney JW Robertson DL Rozanov MN Katz KS Maglott DR Pruitt KD Dieffenbach CW Cataloguing the HIV type 1 human protein interaction network AIDS Res Hum Retroviruses 2008 24 12 1497 1502 10.1089/aid.2008.0113 19025396 PMC2655106

[B41] Ruepp A Brauner B Dunger-Kaltenbach I Frishman G Montrone C Stransky M Waegele B Schmidt T Doudieu ON Stümpflen V Mewes HW CORUM: the comprehensive resource of mammalian protein complexes Nucleic Acids Res 2008 36 Database D646 650 17965090 10.1093/nar/gkm936 PMC2238909

[B42] Tastan O Qi Y Carbonell JG Klein-Seetharaman J Prediction of interactions between HIV-1 and human proteins by information integration Pac Symp Biocomput 2009 516 527 19209727 PMC3263379

[B43] Chatr-Aryamontri A Zanzoni A Ceol A Cesareni G Searching the protein interaction space through the MINT database Methods Mol Biol 2008 484 305 317 10.1007/978-1-59745-398-1_20 18592188

[B44] Yu X Yu Y Liu B Luo K Kong W Mao P Yu X-FF Induction of APOBEC3G ubiquitination and degradation by an HIV-1 Vif-Cul5-SCF complex Science 2003 302 5647 1056 1060 10.1126/science.1089591 14564014

[B45] He N Liu M Hsu J Xue Y Chou S Burlingame A Krogan NJ Alber T Zhou Q HIV-1 Tat and host AFF4 recruit two transcription elongation factors into a bifunctional complex for coordinated activation of HIV-1 transcription Mol Cell 2010 38 3 428 438 10.1016/j.molcel.2010.04.013 20471948 PMC3085314

[B46] Sobhian B Laguette N Yatim A Nakamura M Levy Y Kiernan R Benkirane M HIV-1 Tat assembles a multifunctional transcription elongation complex and stably associates with the 7SK snRNP Mol Cell 2010 38 3 439 451 10.1016/j.molcel.2010.04.012 20471949 PMC3595998

[B47] Kumar D Nath L Kamal MA Varshney A Jain A Singh S Rao KVS Genome-wide analysis of the host intracellular network that regulates survival of Mycobacterium tuberculosis Cell 2010 140 5 731 743 10.1016/j.cell.2010.02.012 20211141

[B48] Paoletti AC Parmely TJ Tomomori-Sato C Sato S Zhu D Conaway RC Conaway JW Florens L Washburn MP Quantitative proteomic analysis of distinct mammalian Mediator complexes using normalized spectral abundance factors Proc Natl Acad Sci USA 2006 103 50 18928 18933 10.1073/pnas.0606379103 17138671 PMC1672612

[B49] Takagi Y Calero G Komori H Brown JA Ehrensberger AH Hudmon A Asturias F Kornberg RD Head module control of mediator interactions Mol Cell 2006 23 3 355 364 10.1016/j.molcel.2006.06.007 16885025

[B50] Cai G Imasaki T Takagi Y Asturias FJ Mediator structural conservation and implications for the regulation mechanism Structure 2009 17 4 559 567 10.1016/j.str.2009.01.016 19368889 PMC2673807

[B51] Cai G Imasaki T Yamada K Cardelli F Takagi Y Asturias FJ Mediator head module structure and functional interactions Nat Struct Mol Biol 2010 17 3 273 279 10.1038/nsmb.1757 20154708 PMC2925518

[B52] Jager S Gulbahce N Cimermancic P Kane J He N Chou S D'Orso I Fernandes J Jang G Frankel AD Alber T Zhou Q Krogan NJ Purification and characterization of HIV-human protein complexes Methods 2011 53 13 19 10.1016/j.ymeth.2010.08.007 20708689 PMC3076283

[B53] Wu J Vallenius T Ovaska K Westermarck J Mäkelä TP Hautaniemi S Integrated network analysis platform for protein-protein interactions Nat Methods 2009 6 1 75 77 10.1038/nmeth.1282 19079255

[B54] Szklarczyk D Franceschini A Kuhn M Simonovic M Roth A Minguez P Doerks T Stark M Muller J Bork P Jensen LJ Mering CV The STRING database in 2011: functional interaction networks of proteins, globally integrated and scored Nucleic Acids Res 2011 39 D561 D568 10.1093/nar/gkq973 21045058 PMC3013807

[B55] Shannon P Markiel A Ozier O Baliga NS Wang JT Ramage D Amin N Schwikowski B Ideker T Cytoscape: a software environment for integrated models of biomolecular interaction networks Genome Res 2003 13 11 2498 2504 10.1101/gr.1239303 14597658 PMC403769

[B56] Suderman M Hallett M Tools for visually exploring biological networks Bioinformatics 2007 23 20 2651 2659 10.1093/bioinformatics/btm401 17720984

[B57] Ceol A Chatr Aryamontri A Licata L Peluso D Briganti L Perfetto L Castagnoli L Cesareni G MINT, the molecular interaction database: 2009 update Nucleic Acids Res 38 Database D532 539 19897547 10.1093/nar/gkp983 PMC2808973

[B58] Breitkreutz BJ Stark C Tyers M Osprey: a network visualization system Genome Biol 2003 4 3 R22 10.1186/gb-2003-4-3-r22 12620107 PMC153462

[B59] Salwinski L Eisenberg D The MiSink Plugin: Cytoscape as a graphical interface to the Database of Interacting Proteins Bioinformatics 2007 23 16 2193 2195 10.1093/bioinformatics/btm304 17553858

[B60] Hernandez-Toro J Prieto C De las Rivas J APID2NET: unified interactome graphic analyzer Bioinformatics 2007 23 18 2495 2497 10.1093/bioinformatics/btm373 17644818

[B61] Driscoll T Dyer MD Murali TM Sobral BW PIG--the pathogen interaction gateway Nucleic Acids Res 2009 37 Database D647 650 18984614 10.1093/nar/gkn799 PMC2686532

[B62] Lin F-KK Pan C-LL Yang J-MM Chuang T-JJ Chen F-CC CAPIH: a Web interface for comparative analyses and visualization of host-HIV protein-protein interactions BMC Microbiol 2009 9 164 10.1186/1471-2180-9-164 19674441 PMC2782265

[B63] Macpherson JI Pinney JW Robertson DL JNets: exploring networks by integrating annotation BMC Bioinformatics 2009 10 95 10.1186/1471-2105-10-95 19323810 PMC2674432

