# Supplementary material for: GPS-Prot: A web-based visualization platform for integrating host-pathogen interaction data
Source: BMC Bioinformatics. 2011 Jul 22;12:298. doi: 10.1186/1471-2105-12-298 (PMC3213248; doi:10.1186/1471-2105-12-298)
Supplement: Additional file 2 [file 1471-2105-12-298-S2.DOC]

# Additional Methods

## GPS-Prot

HIV-human interactions are taken from several sources, with different curation standards: the NIAID HIV1-Human Interaction Database (HHPID) [1], BIND [2] and VirusMINT [3]. Experimental descriptors for HIV-human interactions are available from VirusMINT, which is PSI-MI standardized, and also BIND, which is not standardized. Hence the BIND interactions do not always supply full experimental information. A subset of HHPID is also curated in BIND and VirusMINT and those interactions can be linked to the appropriate experiment descriptors through the same PMID identifiers. However the rest of the interactions in the HHPID database can only be described using the supplied keyword. For these reasons, VirusMINT is our default choice for HIV-human interactions in GPS-Prot as it is fully standardized for information. Users have the option to search the NIAID HHPID database using only a subset of keywords (“Binds”, “Interacts With”, and “Complexes With”) or the full database. For each possible combination of databases, the interaction information and score is based on the most informative entry. When duplicate entries exist for the same interaction, VirusMINT takes precedence over BIND and/or HHPID; and BIND takes precedence over HHPID entries.

Proteins from these databases viewed in GPS-Prot networks sometimes do not directly interact with each other, even though they appear to in the network representation. Indeed, the HHPID estimates that 68% of their interactions are indirect, while only 32% are direct [1]. Thus, whether interactions are direct or indirect cannot be definitively determined based on whether or not an edge appears between two proteins in GPS-Prot. Users should verify interactions of interest using links provided to the comprehensive primary literature for each PPI. The choice of HHPID keywords is also important. For example, neither the VirusMINT database nor our “HHPID: KW=binds, complexes with, interacts with” subset includes interactions assigned the keyword “cleaves”, so substrates of protease do not appear in these networks, despite the fact that they must bind to the enzyme to be cleaved. The same holds true for other reactions such as methylation, phosphorylation, ubiquitination, etc. In order to see all such interactions, therefore, users must select the HHPID (All Keywords) checkbox in GPS-Prot, which will be the most complete set of potential binding partners, but also the noisiest networks.

When using the CORUM database, subunits are always shown linked. That is, in order to cluster proteins in CORUM complexes, we assume an edge between all members of the complex, for visual clarity. For large assemblies, it is unlikely that all subunits contact each other. Other databases similarly contain “binary interactions” between proteins that may not reflect direct physical contacts, but may be part of larger assemblies. CORUM complexes are also always assigned a score of 1, so that they will appear in all networks, despite filtering on the experimentally observed interactions.

Interactions in GPS-Prot are assigned a score. The score is calculated as S=1-a^-x^, where a is 1.3 (chosen to give appropriate dynamic range of scoring) and x is calculated as a sum, Σp_i_, over all publications reporting the interaction. Each publication is assigned a value p_i_, according to the highest-confidence experimental method reported in the paper/database entry (1=Unspecified or Other; 3=SPR, EM/ET, Reconstituted *in vitro* from purified components; 5=3D Structure (x-ray or NMR)). For predicted interactions, the p_i_ value is 0.1 of the Z score [4]. Duplicate publications (e.g. curated in more than one database) are scored only once.

## RNAi-based analysis of early HIV replication stages.

Indicated siRNAs were arrayed in a 384-well plate in triplicate, and assayed for effects on HIV replication (pNL43-Luc-E-R+ pseudotyped with VSVG) and cellular toxicity, as previously described [5] [6]. In addition, 10 scrambled negative control siRNAs were added to the plate as well as siRNAs targeting specific genes that serve as positive control (GL3 luciferase, TNPO3 for viral replication assay and RPS27a for toxicity assay).

siRNA sequences:

| MED30 siRNA1 | CTACAGGATAATCTTCGCCAA |
| --- | --- |
| MED30 siRNA2 | CTGAGATTGGTATATGACAAA |
| MED30 siRNA3 | ATGAAAGATTATTGTAATAAA |
| TNPO3 siRNA1 | ACCGAATGTCTTAGTGAACTA |
| TNPO3 siRNA2 | AACTTCATGGCTAACAATAAA |
| GL3 luciferase | CTTACGCTGAGTACTTCGA |
| RPS27a | AAGCUGGAAGAUGGACGUACU |

## Analysis of mRNA expression levels for screens

Expression data from the UA133 chip [7] were downloaded through BioGPS [8]. This dataset consists of expression values for 13,723 unique human genes, measured across 79 tissues. We took the median expression level for each gene across all tissues and compared the relative expression of genes in the RNAi datasets and the full genome. We repeated this analysis with genes corresponding to complexes in CORUM. The results show that the CORUM database is generally composed of proteins that represent highly expressed transcripts (not shown). We repeated this analysis with transcripts of hits from the HIV genetic screens as compared to the Mtb screen [9] and found that the expression levels are similar (Mann-Whitney U test) (Additional file 6; Figure S1.doc).

## References

1. Ptak RG, Fu W, Sanders-Beer BE, Dickerson JE, Pinney JW, Robertson DL, Rozanov MN, Katz KS, Maglott DR, Pruitt KD, Dieffenbach CW: **Cataloguing the HIV type 1 human protein interaction network.** *AIDS Res Hum Retroviruses* 2008, **24**(12):1497-1502.

2. Alfarano C, Andrade CE, Anthony K, Bahroos N, Bajec M, Bantoft K, Betel D, Bobechko B, Boutilier K, Burgess E, Buzadzija K, Cavero R, D'Abreo C, Donaldson I, Dorairajoo D, Dumontier MJ, Dumontier MR, Earles V, Farrall R, Feldman H, Garderman E, Gong Y, Gonzaga R, Grytsan V, Gryz E, Gu V, Haldorsen E, Halupa A, Haw R, Hrvojic A *et al*: **The Biomolecular Interaction Network Database and related tools 2005 update**. *Nucleic Acids Res* 2005, **33**(Database issue):D418-424.

3. Chatr-aryamontri A, Ceol A, Peluso D, Nardozza A, Panni S, Sacco F, Tinti M, Smolyar A, Castagnoli L, Vidal M, Cusick ME, Cesareni G: **VirusMINT: a viral protein interaction database.** *Nucleic Acids Res* 2009, **37**(Database issue):D669-673.

4. Tastan O, Qi Y, Carbonell JG, Klein-Seetharaman J: **Prediction of interactions between HIV-1 and human proteins by information integration.** *Pac Symp Biocomput* 2009:516-527.

5. König R, Chiang C-yY, Tu BP, Yan SF, DeJesus PD, Romero A, Bergauer T, Orth A, Krueger U, Zhou Y, Chanda SK: **A probability-based approach for the analysis of large-scale RNAi screens.** *Nat Methods* 2007, **4**(10):847-849.

6. König R, Zhou Y, Elleder D, Diamond TL, Bonamy GMC, Irelan JT, Chiang C-YY, Tu BP, De Jesus PD, Lilley CE, Seidel S, Opaluch AM, Caldwell JS, Weitzman MD, Kuhen KL, Bandyopadhyay S, Ideker T, Orth AP, Miraglia LJ, Bushman FD, Young JA, Chanda SK: **Global analysis of host-pathogen interactions that regulate early-stage HIV-1 replication.** *Cell* 2008, **135**(1):49-60.

7. Su AI, Wiltshire T, Batalov S, Lapp H, Ching KA, Block D, Zhang J, Soden R, Hayakawa M, Kreiman G, Cooke MP, Walker JR, Hogenesch JB: **A gene atlas of the mouse and human protein-encoding transcriptomes.** *Proc Natl Acad Sci U S A* 2004, **101**(16):6062-6067.

8. Wu J, Vallenius T, Ovaska K, Westermarck J, Mäkelä TP, Hautaniemi S: **Integrated network analysis platform for protein-protein interactions.** *Nat Methods* 2009, **6**(1):75-77.

9. Kumar D, Nath L, Kamal MA, Varshney A, Jain A, Singh S, Rao KVS: **Genome-wide analysis of the host intracellular network that regulates survival of Mycobacterium tuberculosis.** *Cell* 2010, **140**(5):731-743.
