# Supplementary material for: GPS-Prot: A web-based visualization platform for integrating host-pathogen interaction data
Source: BMC Bioinformatics. 2011 Jul 22;12:298. doi: 10.1186/1471-2105-12-298 (PMC3213248; doi:10.1186/1471-2105-12-298)
Supplement: Additional file 6 — Comparison of broad expression level of Mtb and HIV screens. [file 1471-2105-12-298-S6.DOC]

**
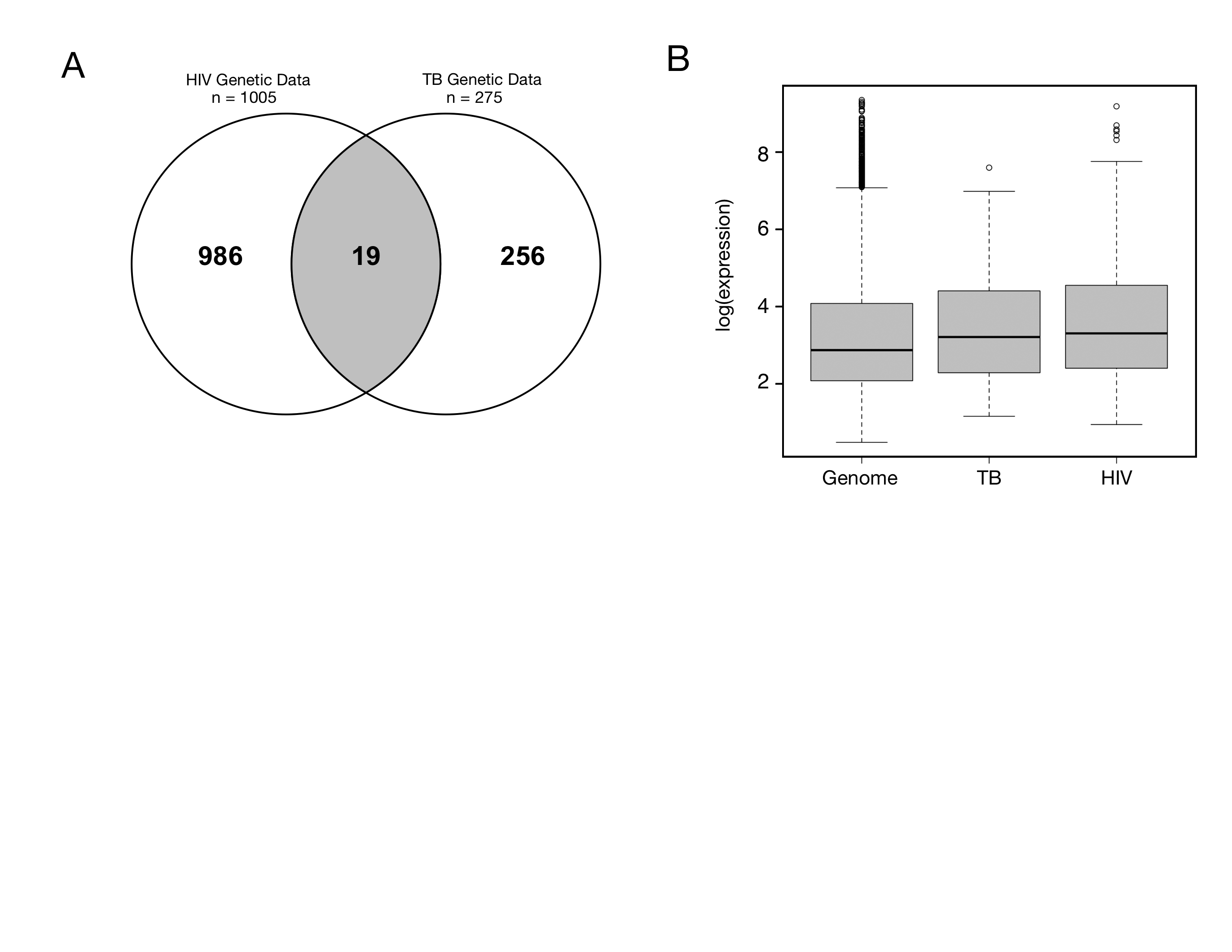
**

**Figure S1**. Comparison of broad expression level of Mtb and HIV screens

Box plots of mRNA transcript expression levels as measured by Su et al. (*Su et* al, 2004) and analyzed for different screens as described in Additional file 2 (see Additional file 2: Additional_methods.doc). Expression level for each gene is taken as the median value across all tissues measured. We do not observe a significant difference in the median expression levels of mRNA transcripts identified in either the HIV or Mtb genetic screens, (p = 0.6837).
