# Supplementary material for: GPS-Prot: A web-based visualization platform for integrating host-pathogen interaction data
Source: BMC Bioinformatics. 2011 Jul 22;12:298. doi: 10.1186/1471-2105-12-298 (PMC3213248; doi:10.1186/1471-2105-12-298)
Supplement: Additional file 7 — RNAi-mediated depletion of MED30 blocks early steps of replication of a VSV-G pseudotyped HIV luciferase virus. [file 1471-2105-12-298-S7.DOC]

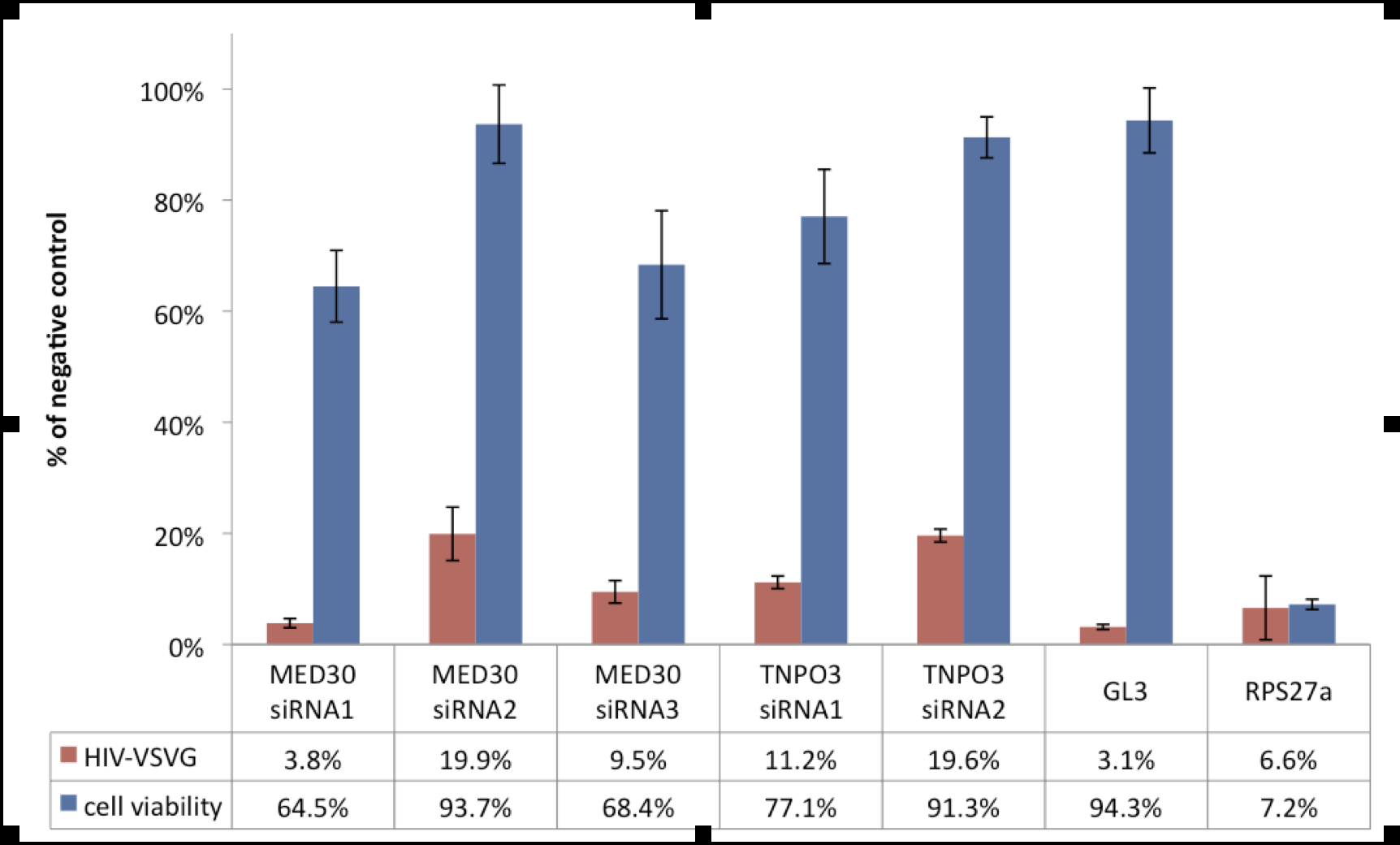


**Figure S2**. RNAi-mediated depletion of MED30 blocks early steps of replication of a VSV-G pseudotyped HIV luciferase virus.

Three unique siRNAs targeting MED30 were found to inhibit early stage viral replication in human 293T cells, while not inducing significant effects on cell viability. siRNAs targeting TNPO3 (Kön*ig et* al, 2008) (Bra*ss et* al, 2008), GL3 luciferase and RPS27a served as controls for the viral replication assay and the cell viability assay, respectively. Values represent means and standard deviations of three experiments.
